# Editorial: Prescribing Psychotropics: Misuse, Abuse, Dependence, Withdrawal and Addiction

**DOI:** 10.3389/fpsyt.2021.688434

**Published:** 2021-04-30

**Authors:** Stefania Chiappini, Fabrizio Schifano, Giovanni Martinotti

**Affiliations:** ^1^Psychopharmacology, Drug Misuse and Novel Psychoactive Substances Research Unit, School of Life and Medical Sciences, University of Hertfordshire, Hatfield, United Kingdom; ^2^Department of Neuroscience, Imaging and Clinical Sciences, “G. D'Annunzio” University, Chieti, Italy

**Keywords:** drug abuse, drug misuse, prescription drug misuse, pharming, drug diversion, addiction, pharmacovigilance

Over the last decade, the trend of drug consumption has changed dramatically. The advent of a high number of new psychoactive substances (NPS) has contributed to the appearance and growth of a new “drug scenario” (1, 2) characterized by an increasing number of molecules with unknown effects; poor safety profiles and acute drug toxicity presentations; and psychiatric consequences (3–6). In this context, medications' misuse appears to be an increasingly concerning phenomenon, specifically driven by the already recorded rise in the opioid use, benzodiazepines, and other Central Nervous System (CNS) depressants (including sedatives and hypnotics), and prescription stimulants, e.g., amphetamines, methamphetamines, methylphenidate (7, 8). However, a range of remaining molecules have been reported as being misused; diverted; and recorded by drug users' online websites suggesting new trends and experimentations specifically with medicinal compounds (9–11). An increasing awareness regarding these issues has been contributing to the development of pharmacovigilance studies regarding the possible potential of misuse/abuse/dependence and withdrawal of both prescription (e.g., quetiapine, gabapentinoids, olanzapine, bupropion, etc.) and over-the-counter (OTC) drugs (e.g., loperamide, dextromethorphan, promethazine, benzydamine etc.) (12–17). Indeed, pharmacovigilance studies have helped in identifying signals of misuse associated with these molecules (18). For instance, whilst both pregabalin and gabapentin are approved treatments for epilepsy and neuropathic pain disorders, with pregabalin being prescribed as well in some countries for the treatment of generalized anxiety disorder (19), they have increasingly been reported for their misusing potential, especially when used in combination with opioids and sedatives (12). In 2018, after safety warnings following an increase in deaths related to their use, the UK Advisory Council on the Misuse of Drugs (ACMD) recommended that both had to be controlled under the Misuse of Drugs Act 1971 as Class C substances and scheduled under the Misuse of Drugs Regulations 2001 as Schedule 3, so as not to preclude legitimate use on prescription (20). Conversely, a range of factors are thought to contribute to the non-medical use of prescription/OTC drugs, such as the perception of these molecules being more socially acceptable/less stigmatizing; likely lack of detection in standard drug screens; and safer than remaining illicit substances as well. “Pharming”; “pharm-parties”; and “doctor-shopping” attitudes, involving high-/mega-dosage prescription drugs' intake, are trends which are increasingly being reported among young adult populations (9, 21, 22). In parallel with this, increasing levels of access to the web over the past 15 years or so may have boosted the current scenario of prescribed drugs' misuse and abuse, with social networks playing a role in medications' aggressive marketing/distribution from rogue “pharmacy” websites (10, 23–25). Moreover, the web has been contributing to the diffusion of new synthetic compounds, such as designer benzodiazepines and illicit fentanyl analogs, which are associated with a high abuse potential and severe adverse effects including coma and death (26–28). Finally, since the beginning of 2020, due to drug shortage issues resulting to the COVID-19 pandemic, a shift in misusing behavior relating to both prescription and OTC medicines has been recorded (10, 13, 29–33).

Consistent with these issues, the current Research Topic has focussed on the assessment of the misuse, abuse, dependence, withdrawal, diversion, and addiction potential of prescribing and OTC drugs. A range of original research papers, systematic reviews, meta-analysis, reviews, and case reports are here made available. This Research Topic will hopefully shed further light on the harms associated with medications' misuse and abuse, highlighting the importance of this field for clinicians; prescribers; and health professionals in general. Indeed, 13 original articles of excellent quality and likely broad impact are here offered to the Frontiers in Psychiatry readers. A description of prescription drugs' misuse in “clubbers” and disco goers in Ibiza showed that current trends of such phenomenon may not be limited to subjects with psychiatric disorders, as prescription drugs may be used an alternative to classic and novel psychoactive compounds and/or may be used to tamper and self-medicate the effects determined by the use of substances. Considering prescription drugs misused, the diversion of the benzodiazepine etizolam was here recorded, being characterized by high-dosage intake and resulting dependence issues; also, the misuse and diversion of several OTCs, including antihistamines (e.g., diphenhydramine, promethazine, chlorpheniramine, and dimenhydrinate); dextromethorphan- and codeine-based cough medicines; and the nasal decongestant pseudoephedrine have here been reported. Furthermore, a few surveys are here being collected; the first one is a European survey investigating psychiatry trainees' attitudes, knowledge and training in addiction psychiatry, while a second paper evaluated the German addiction medicine physicians' knowledge of both health and psychosocial harms of 33 psychoactive substances, including opioids and non-opioid prescription analgesics, e.g., gabapentinoids. Finally, using data from the RADARS® survey on the non-medical use of prescription drugs conducted in five European countries, the non-medical use of gabapentinoids resulted to have the highest prevalence in Germany and UK compared with Spain, Italy, and France. Data related to gabapentinoids as recorded by the French Addictovigilance Network confirmed the importance of pharmacovigilance monitoring for gabapentinoids due to their abuse potential and their related health harms, including hospitalization for serious neurologic, psychiatric or cardiac effects; requests for specific support; and deaths. Similarly, the analysis of the FDA Adverse Event Reporting System (FAERS) database, using big data search analytics as a supplementary tool to detect drug abuse-related safety signals, supported these issues. In parallel, within a multidimensional monitoring of prescription drug abuse, the early detection and quantification of “doctor shopping” practices may well need to be considered essential. Moreover, the identification of specific personality traits (e.g., hopelessness, anxiety sensitivity, sensation seeking, and impulsivity) and psychometric indicators (e.g., the Severity of Dependence Scale- SDS) might be useful in providing drug abusers with personalized interventions and strategies. Finally, the treatment of drug intoxication, as in a case of kratom use disorder, and of drug withdrawal through the continuous infusion of flumazenil in the management of benzodiazepines detoxification were here described.

In conclusion, the abuse of prescription and OTC drugs has become an issue of increasing public concern across the globe (34). Whilst health services are already under unprecedented levels of strain, the current drug scenarios have further modified, in parallel with the current pandemic-related goods' and people local; national; and international restrictions of movements. At these challenging times, healthcare professionals are recommended to both be vigilant and develop strategies to ensure continuity of care for people who use drugs and people with drug use disorders, whilst preventing as well possible medicines' misuse and diversion issues (9).

## Author Contributions

All authors listed have made a substantial, direct and intellectual contribution to the work, and approved it for publication.

## Conflict of Interest

FS was a member of the UK Advisory Council on the Misuse of Drugs (ACMD; 2011–2019) and is currently a member of the EMA Advisory Board (Psychiatry). GM has been a consultant and/or a speaker and/or has received research grants from Angelini, Doc Generici, Janssen-Cilag, Lundbeck, Otsuka, Pfizer, Servier, Recordati. The remaining author declares that the research was conducted in the absence of any commercial or financial relationships that could be construed as a potential conflict of interest.
